# Watch-and-Wait as a Therapeutic Strategy in Rectal Cancer

**DOI:** 10.1007/s11888-018-0398-5

**Published:** 2018-03-07

**Authors:** Laurence Bernier, Svetlana Balyasnikova, Diana Tait, Gina Brown

**Affiliations:** 1 CHU de Québec – Hôtel-Dieu de Québec, 11 côte du Palais, Québec, QC G1R 2J6 Canada; 2 The Royal Marsden NHS Foundation Trust, Downs Road, Sutton, Surrey, SM2 5PT UK; 3 Department of Clinical Oncology, The Royal Marsden NHS Foundation Trust, Downs Road, Sutton, Surrey, SM2 5PT UK; 4 Gastrointestinal Cancer Imaging, Department of Radiology, The Royal Marsden NHS Foundation Trust NIHR BRC and Imperial College London, Downs Road, Sutton, Surrey, SM2 5PT UK

**Keywords:** Watch and wait, Deferral of surgery, Non-operative management, Complete response, Rectal cancer, Organ preservation

## Abstract

**Purpose of Review:**

Pathological complete response is seen in approximately one fifth of rectal cancer patients following neoadjuvant chemoradiation. Since these patients have excellent oncological outcomes, there has been a rapidly growing interest in organ preservation for those who develop a clinical complete response. We review the watch-and-wait strategy and focus on all aspects of this hot topic, including who should be considered for this approach, how should we identify treatment response and what are the expected outcomes.

**Recent Findings:**

The major challenges in interpreting the data on watch-and-wait are the significant heterogeneity of patients selected for this approach and of methods employed to identify them. The evidence available comes mostly from retrospective cohort studies, but has shown good oncological outcomes, including the rate of successful salvage surgery, locoregional control and overall survival.

**Summary:**

There is currently not enough and not robust enough evidence to support watch-and-wait as a standard approach, outside a clinical trial, for patients achieving clinical complete response following neoadjuvant chemoradiation. Furthermore, there is a lack of data on long-term outcomes. However, the results we have so far are promising, and there is therefore an urgent need for randomised control studies such as the TRIGGER trial to confirm the safety of this strategy.

## Introduction

The standard treatment for high-risk non-metastatic rectal adenocarcinoma is neoadjuvant chemoradiation (nCRT) followed by total mesorectal excision (TME) with or without adjuvant chemotherapy. This strategy provides good oncological outcomes, with a rate of local relapse of less than 10% [1, 2]. However, TME is a radical surgical procedure with a significant risk of perioperative morbidities, including bowel, sexual and urinary dysfunction [3–7]. Moreover, in advanced low rectal cancers, the outcome of treatment frequently results in permanent stoma formation.

We have known for a long time now that radiotherapy can cure cancer. Nigro and his team paved the way in the early 1970s, believing in the role of definitive chemoradiation in squamous cell carcinoma of the anal canal [8]. It took several years to build enough evidence to support a radical change of practice, but it is now the standard management and surgery is considered a salvage procedure. In rectal cancer, it is not reasonable to hope for the same outcome, the main reason being the difference in sensitivity to chemoradiation between adenocarcinoma and squamous cell carcinoma. Nevertheless, pathological complete response (pCR) is seen in 10–25% of rectal cancers following nCRT [9]. Angelita Habr-Gama, like Norman Nigro, has pioneered a similar approach and has defended for more than a decade a non-operative management in rectal cancer patients who achieved a good response to chemoradiation [10]. The oncological community was sceptical at first, but now interest is growing fast. The watch-and-wait (W&W) approach is attractive, both to patients and clinicians. It has shown excellent outcomes, as long as candidates are carefully selected and appropriately monitored. Below is a review of this management strategy, focusing on the latest advances and future directions.

## Overview and Background

In 2004, Habr-Gama and her team, from São Paulo in Brazil, published their first long-term outcomes of a cohort of patients managed with a non-operative strategy after having achieved a clinical complete response (cCR) following nCRT [10]. Two hundred and sixty-five patients with resectable distal rectal cancer received standard nCRT. Patients were reassessed at 8 weeks following treatment, using clinical, radiological and endoscopic examinations. Those with a cCR avoided immediate surgery and entered a strict surveillance program. Seventy-one patients (26.8%) obtained cCR and formed this observation group. With a median follow-up of 57.3 months, only two patients had local tumour regrowth, and three patients developed a systemic recurrence. The 5-year overall survival and disease-free survival rates were 100 and 92%, respectively. This landmark study revealed an entire new concept in rectal cancer management and indicated the potential for organ preservation. Since then, the results have been updated, the treatment schedule has changed and many cancer centres are now sharing their own results and helping to build the evidence. Despite this, still many aspects of the W&W approach are undefined or controversial.

## Patient Selection for Preoperative Therapy

The main challenge with W&W is the selection of patients who can be considered for this approach. The evidence available comes mainly from retrospective data, with great variations on who has been offered surveillance, including patients characteristics and tumour stage. Furthermore, some studies have used inaccurate and insufficient staging modalities, and others have not even reported on tumour staging at baseline. This heterogeneity limits the interpretation of data.

### Patients Not Fit for Radical Resection

There are patients whose comorbidities or performance status preclude any attempt at radical operation, who are instead offered CRT or short-course radiotherapy as an alternative for definitive treatment. Some of them appear to achieve a cCR and are being subsequently monitored, even though salvage radical surgery will never be an option in case of tumour regrowth. These patients form an entirely different entity and should be excluded from trials involving W&W. The essence of active surveillance is in patients who are able to avoid surgery, but to whom the procedure will be considered at a time when we feel we need to intervene.

### Patients Who Wish to Avoid Abdominoperineal Excision (APE) at Any Cost

A large number of patients end up being monitored because they decline radical surgery. Most of the time, they receive long-course CRT with or without local excision, and those who achieve a cCR or nearCR enter a surveillance program. These patients represent a significant proportion of patients in the retrospective studies on W&W. Unfortunately, their baseline characteristics are often poorly recorded, making it difficult to know if they would have normally been offered nCRT on a basis of high-risk tumour at baseline. At present, it is not possible to advise a patient who is facing an APE what the likelihood is that preoperative CRT will succeed in achieving a CR and thus the likely success of a W&W approach. Since the pCR rates have been reported to be approximately 25% at best, it is therefore logical to conclude that additional RT where it is not indicated in the hope of achieving CR may not succeed in up to 75% of patients.

### Patients with Early-Stage Low Tumours

Patients with early-stage low rectal tumours, without any adverse features, amenable to local excision or transanal endoscopic microsurgery should undergo the procedure without neoadjuvant treatment. If histopathology confirms the full excision and initial low risk preoperative staging, there is no indication for further treatment, and this approach provides excellent oncological and functional outcomes. However, when high risk features are present, such as third submucosal layer (sm3) invasion, positive margin, grade 3, lymphovascular invasion, tumour budding or mucinous subtype, and in cases of pT2 tumours, local resection alone is not sufficient. Local and/or regional recurrences can be seen in as much as 20% of cases [11]. The standard treatment for completion is radical resection, and since the tumours are low, in the majority of cases it results in a permanent stoma. In the context of organ preservation, only few studies have included patients with early-stage tumours. Habr-Gama et al. did a retrospective study assessing the outcomes of a W&W strategy on patients with cT2N0 rectal adenocarcinoma, less than 7 cm from the anal verge, following two different regimen of nCRT [12•]. Patients in the ‘extended’ CRT group (54Gy of radiation and 6 cycles of 5-fluorouracil (5FU)-based chemotherapy) were more likely to achieve a cCR compared to the standard group (50.4 Gy and 2 cycles of 5FU-based chemotherapy) (85.7 vs 56.6%, *p* < 0.001). As mentioned above, in the initial study by Habr-Gama [10], the rate of cCR was 26.8% for the entire cohort, which comprised T3 tumours in 69% of cases. Therefore, achieving a rate of cCR of 85%, or even 56% with a standard regimen of nCRT, is impressive and suggests the potential of early tumours for W&W. Similarly, an Italian group of investigators have cumulated several years of experience in organ preservation in early-stage low rectal cancers. They have reported in 2012 the results of a prospective randomised clinical trial comparing endoluminal locoregional resection (ELRR) with laparoscopic total mesorectal excision (TME) for early-stage cancers following nCRT [13]. Eligible patients had clinically staged T2N0M0 disease (staging modalities including endorectal ultrasonography, sigmoidoscopy with biopsies, pelvic MRI and whole-body CT), located within 6 cm of the anal verge, grade 1 or 2 tumours with a diameter of less than 3 cm. Patients with lymphovascular or perineural invasion were excluded. The patients in the study received upfront nCRT (50.4 Gy in 5 weeks with concomitant 5FU), had restaging investigations 6 weeks after treatment and were then randomised between ELRR and TME. With a median follow-up of 9.6 years, there was no statistically significant difference between the two groups with regards to locoregional recurrences, rate of distant metastases, cancer-specific survival, disease-free survival and overall survival. The rate of pCR with nCRT was approximately 27% (28% in ELRR group, 26% in TME group). Other studies investigated local excision following nCRT in early-stage low rectal cancer and reported higher rates of pCR (49–59%) [14•, 15, 16]. All these studies provide interesting data in favour of nCRT followed by surveillance or local resection in patients with early-stage low cancers, suggesting that these patients might actually be ideal candidates for organ preservation. However, they involve a relatively small number of patients. The STAR-TREC trial (NCT02945566) is ongoing and should help clarify the question. Although promising, this subject raises an ethical issue. Unless there is a threatened surgical resection margin, which is considered as a high risk feature, patients with early-stage rectal cancer are not typically considered for neoadjuvant treatment. Offering upfront CRT to these patients, the decision based purely on clinical staging, is risky. At our institution, the initial staging of early low rectal cancer includes a high-resolution pelvic MRI amongst other standard investigations. The results are discussed in our weekly multidisciplinary meeting where images are reviewed and management options discussed. When the CRM is clear and the tumour amenable to local resection, we tend to favour this approach as first-line intervention and rediscuss the case with the final histopathology report. It has the advantage of providing accurate pathological staging on which to base future management decisions. When further treatment is recommended, we favour discussion with the patients regarding completion radical surgery versus CRT in an attempt at organ preservation with close imaging and clinical surveillance, knowing that the latter is not yet standard. Although we have reported previously on the good oncological outcomes of patients who decline surgery and undergo adjuvant CRT [17], we prefer, if available, participation in research trials.

### Patients with High-Risk Low Tumours

In rectal cancer, an incomplete resection is associated with a higher risk of local recurrence and significantly worse outcomes [18, 19]. The rate of R1 resection was traditionally reported as high as 20–40% for low rectal tumours, partly explained by the complexity of the surgical technique in such a confined anatomical space [20–22]. The MERCURY II trial, investigating specifically low rectal tumours, validated an MRI staging classification to document the relationship between the low rectal cancer surgical plane (mrLRP) and the tumour [23••]. The overall rate of pCRM involvement in the study was less than 10%, proving the value of mrLRP radiological assessment. Furthermore, 4 factors were found on multivariate regression to be predictive of pCRM involvement: an unsafe mrLRP, invasion from the tumour of the anterior quadrant of the rectum, a tumour height of less than 4 cm from the anal verge and the presence of extramural vascular invasion on MRI (mrEMVI). With these factors, the study group was able to propose a predicted risk of pCRM involvement. Tumours with none of the bad features mentioned above have a risk of involved pCRM of 1%, whereas the risk is as high as 60% with tumours presenting all four factors. Based on this data, nCRT is justified in patients with high-risk low tumours. However, as emphasised in the MERCURY II study, restaging investigations, including MRI and reassessment of the surgical plane is mandatory after nCRT. Patients who achieve a cCR or nearCR could be offered organ preservation. In patients for whom surgery is inevitable, a ymrLRP MRI assessment is crucial, as an unsafe plane carries a 25% risk of an incomplete resection. These patients might therefore be candidates for intensified treatment, such as further consolidation chemotherapy or surgery beyond TME with excision of the compartments involved by the treated tumour.

### Patients with Advanced Cancer

Patients with locally advanced rectal cancer typically require nCRT, for its proven benefit on locoregional control [2]. Those who reach a cCR following treatment could be candidates for W&W. We have previously discussed the low rectal tumours, but what about the proximal ones, those tumours for which a low anterior resection with sphincter preservation is feasible? There are some pros and cons of a NOM in these cases. Firstly, avoiding surgery prevents, at least temporarily, the patient from facing the possible consequences of a triple-modality treatment on bowel, urinary and sexual function and its impact on quality of life [24]. The low anterior resection syndrome (LARS), which is characterised by symptoms such as faecal incontinence, emptying difficulties, urgencies and fragmented bowel movements, is a recognised entity and is seen in as much as 90% of patients who undergo an anterior resection [25–27]. Several studies have found that nCRT is a significant factor of poorer functional outcomes after surgery. Secondly, a radical procedure such as TME has its risks of surgical complications, morbidities and mortality [6]. On the other hand, one could argue that salvage surgery might potentially be more challenging than an upfront procedure. This has been described in anal cancer, where surgery is a salvage strategy and carries a higher risk of complications [28, 29]. The late side effects of chemoradiation, including pelvic fibrosis, can complexify the surgical technique. Another argument against W&W in higher tumours is the unavailability of digital rectal examination (DRE). Tumours over 6–7 cm from the anal verge cannot be reached with the examining finger, and DRE has been proven a helpful tool in tumour response assessment and monitoring. At our institution, we favour imaging modalities in our monitoring protocol, but agree that DRE should not be completely discarded. The response assessment will be further discussed below. Finally, and possibly the main argument against W&W in the proximal tumours, is the lack of data on long-term outcomes, as they have often been excluded from the studies.

**Fig. 1:**
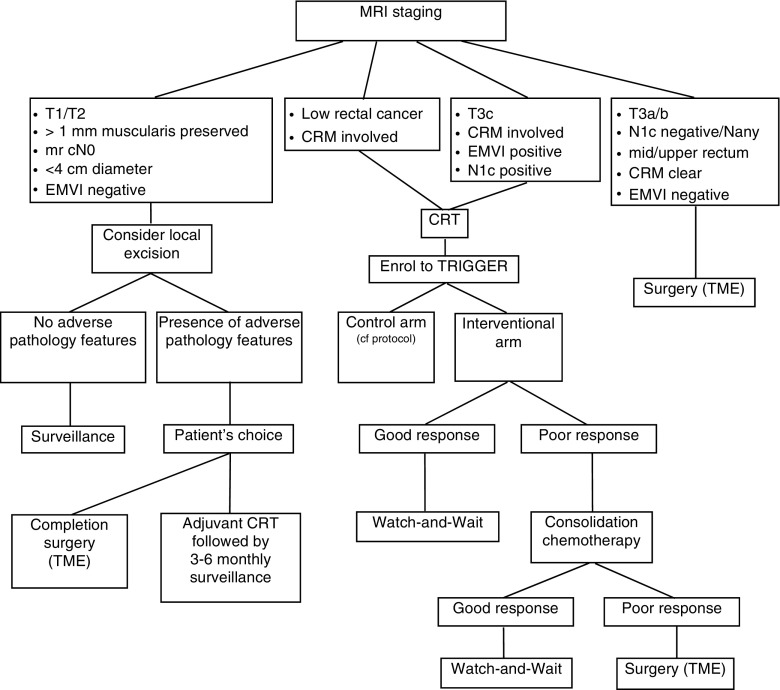
Rectal cancer management at the Royal Marsden Hospital

In summary, when analysing data on W&W, the reader should pay careful attention to the description of the population included (stage of disease, methods of baseline and treatment response assessments, quality of imaging). The limitations of the data we have so far are related to the lack of accurate staging or standardisation of selection policies for CRT. Figure 1 summarises our current institutional policy for rectal cancer management, including organ preservation strategies.

## What Is the Best Treatment—Duration and Intensification of Treatment

Most series on W&W describe long-course chemoradiation as the preferred neoadjuvant approach, with pelvic radiotherapy of 45–54 Gy combined with either 5FU or capecitabine being the most common regimen. However, there are several studies looking at ways to optimise the therapy with the objective of increasing the pCR rate and therefore potentially the rate of organ preservation.

### Standard CRT

In the landmark study by the German Rectal Cancer Study Group, in which patients were randomly assigned to preoperative CRT versus postoperative CRT, the pCR rate in the preoperative group was 8% [30]. The treatment administered was 50.4 Gy of radiotherapy, combined with continuous infusion of 5FU. Other studies, including a meta-analysis from the Cochrane group, have reported rates of pCR of 11–16% [31–33]. Organ preservation using this standard combination of RT and 5FU or Capecitabine has shown good outcomes, which will be discussed further below.

### Short-Course Radiotherapy

There is very little evidence on W&W following short-course radiotherapy. To our knowledge, the only data available comes from a prospective study of organ preservation (W&W or local excision) in elderly patients [34]. In a cohort of 60 patients (age 70 years or older, cT1-4N0-3M0 rectal adenocarcinoma), 30 were not candidates for chemotherapy and were therefore offered short-course radiotherapy (SCRT) with delayed assessment of the tumour response. The investigators presented the results of this unplanned interim subgroup analysis, in which they evaluated the feasibility of SCRT with delayed evaluation of response in a W&W context. The 30 patients in the SCRT group received 5 × 5 Gy over 1 week, and 4 of them had a further 4 Gy boost in 1 fraction, 1 week after completing their radiotherapy. The median interval between the last fraction and the response assessment was 10.3 weeks. Six patients (20%) achieved a cCR, and all entered a W&W surveillance program. With a median follow-up of 21 months, 1 of those 6 patients experience tumour regrowth, and the management and outcome of this specific patient is not described in the article. The Stockholm III trial randomised patients between SCRT followed by surgery within 1 week, SCRT followed by surgery at 4–8 weeks and finally long-course radiotherapy (LCRT) of 50 Gy followed by surgery within 4–8 weeks [35]. Even though organ preservation was not part of this trial, looking at the pCR rate can be informative when discussing treatment strategies in W&W. No information was given about the pCR rate with long-course RT. Surgery at 1 week after SCRT led to a very low rate of pCR of 1.7%, whereas delaying surgery to 4–8 weeks increased it to 11.8%, which is still relatively low [36]. This could indirectly indicate the superiority of long-course chemoradiation in a W&W program.

### Contact X-Ray Brachytherapy

In France (Lyon and Nice), Jean-Pierre Gérard and his team have one of the largest experiences in contact x-ray brachytherapy (CXB) (Papillon technique). The results of their studies have provided strong evidence supporting the use of CXB in selected cases, with high rates of organ preservation and low rates of local recurrence [37, 38]. Sun Myint and colleagues also shared their experience with CXB [39]. They reported on the rate of local regrowth in a context of organ preservation, in 200 patients treated at their centre between 2003 and 2012. Their cohort comprised 17 patients with very early tumours, less than 3 cm, who were offered CXB upfront after declining surgery or being deemed non-surgical candidates. The remaining patients were all patients with diverse tumour stages, who received CRT (45Gy with concomitant 5FU or Capecitabine) or RT alone (dose not reported). These patients had restaging investigations 6–8 weeks following treatment, and those with a residual disease of less than 3 cm were offered further RT with CXB of 90 Gy in 3 fractions. Again, all these patients either declined surgery or were considered unfit for radical resection, hence the referral for further treatment with CXB. A total of 144 patients achieved cCR post CXB, and only 16 of them (11%) experienced local regrowth. The authors argue in favour of CXB for tumour boost, as it has shown one of the lowest reported rates of local regrowth, but these results need to be interpreted cautiously. All these tumours had already shown good regression, as only those showing residual disease less than 3 cm were eligible for CXB. A selection bias might have been introduced, the cohort including potentially only highly radiosensitive and biologically favourable tumours. Another study by Dhadda et al. has also shown a low recurrence rate of 12%, with a similar cohort of patients, but the same biases and critics apply [40]. The Lyon R96-02 trial aimed to compare CXB with external beam radiotherapy (EBRT), but the study was running at the end of the 1990s [41]. The EBRT techniques were not as sophisticated as the current standard, the EBRT alone arm did not include concomitant chemotherapy and the dose delivered (39 Gy in 13 fractions) is no longer recommended. The OPERA study (NCT02505750) is a phase III international randomised trial currently recruiting. Patients with cT2-T3a/b tumours, less than 5 cm, are randomised between two techniques of boost delivery following nCRT: EBRT of 9 Gy in 5 fractions, versus CXB of 90 Gy in 3 fractions. Hopefully, it will provide answers regarding methods of boost delivery and dose escalation.

### Addition of Consolidation Chemotherapy vs Neoadjuvant Chemotherapy

Several groups have studied the intensification or addition of chemotherapy in the neoadjuvant part of the treatment. The most frequently studied, and possibly the most promising, is the addition of oxaliplatin. The CAO/ARO/AIO-04 trial investigated the addition of oxaliplatin, both pre- and postoperatively, to the standard regimen of nCRT [42••]. Patients with cT3-4Nany or cTanyNpositive disease were randomised between two arms. The standard arm received nCRT (50.4 Gy with 5FU) followed by surgery and 4 cycles of bolus 5FU. The interventional arm received the same radiotherapy plus infusional 5FU and oxaliplatin, followed by surgery and 8 cycles of a combination of oxaliplatin, leucovorin and infusional 5FU. The treatment including oxaliplatin was well tolerated, without significantly different side effects than the standard arm. Furthermore, the oxaliplatin arm led to a pCR rate of 17% (104/596 patients), compared to 13% (81/615) in the standard arm (*p* = 0.031). Many other groups have studied the inclusion of oxaliplatin and, in addition to increased toxicities, none has resulted in a statistically significant improvement of the pCR rate [43–46]. However, as mentioned by the authors in the German study (CAO/ARO/AIO-04), the toxicity of the chemotherapy regimen in these studies had a significant impact on the compliance, which could explain the pCR rate.

More recently, the FOWARC trial randomised patients with stage II-III rectal cancer between three arms: nCRT (46–50.4 Gy with 5FU) followed by surgery and adjuvant 5FU, the same regimen with the addition of oxaliplatin (mFOLFOX-6) and finally 4–6 cycles of mFOLFOX-6 followed by surgery and a further 6–8 cycles of mFOLFOX-6 [47•]. The rate of pCR in the standard arm, mFOLFOX-6 + radiotherapy arm and mFOLFOX-6 alone arm was, respectively, 14, 27.5 and 6.6%. Understandably, the toxicities were higher in the mFOLFOX-6 + radiotherapy arm, but surprisingly the compliance was as good if not better than the standard arm group. A group from China performed a meta-analysis on the addition of oxaliplatin to the standard 5FU-based regimen of nCRT [48]. It included, amongst others, the studies mentioned above, and found that the regimen including oxaliplatin significantly increased the rate of pCR (RR = 1.24, 95% CI 1.02–1.51; *p* = 0.03). It also led to an improved disease-free survival and lower rate of distant metastases.

In 2006, the Angelita & Joaquim Gama Institute, in São Paulo, Brazil, changed their routine practice and started offering an ‘extended’ nCRT regimen, which consists of 54 Gy in 30 fractions with concomitant chemotherapy followed by a course of consolidation chemotherapy (5FU and leucovorin for a total of 6 cycles: 3 cycles delivered every 21 days and 3 additional cycles delivered during the resting period after radiotherapy completion). They recently retrospectively compared the outcomes of patients with cT2N0 disease in the standard regimen with the extended regimen and found that the extended regimen, which was well tolerated, led to a significant increase in the cCR rate (56.6 vs 85.7%) [12•]. Therefore, patients who received the extended regimen were more likely to be eligible to a W&W approach.

The Memorial Sloan Kettering Cancer Centre (MSKCC) is currently running a phase II study in which patients are randomised between induction chemotherapy followed by nCRT, and nCRT followed by consolidation chemotherapy (NCT02008656) [49]. Patients with a significant clinical response to treatment are being management with a non-operative strategy.

While some people investigate the intensification of treatment, others are trying to customise it, for example by studying the feasibility of omitting routine use of radiotherapy in locally advanced rectal cancer. The MSKCC has done so in 2014 with a pilot study [50]. Thirty-two patients, with cT2-T3Nany rectal adenocarcinoma received preoperative infusional 5FU, leucovorin and oxaliplatin (FOLFOX) plus bevacizumab. Patients who maintained a stable disease or showed progression proceeded with neoadjuvant radiotherapy prior to radical resection (TME), whereas patients who responded to treatment went straight to surgery (TME). Four patients needed radiotherapy: 2 patients preoperatively and 2 patients postoperatively for positive resection margins. The pCR rate of patients who received chemotherapy alone was 25%. The GEMCAD 0801 trial investigated the safety and efficacy of neoadjuvant chemotherapy alone (capecitabine, oxaliplatin (CAPOX) and bevacizumab) in 46 patients with T3 rectal adenocarcinoma with clear predicted resection margins on MRI, also in that case with a selective use of nCRT for patients with progressive disease only [51, 52]. They achieved similar results as the MSKCC pilot study, with a pCR rate of 20%. Although interesting, this strategy of offering nCRT only to selected patients after an induction course of chemotherapy is not yet an approved treatment option outside of a clinical trial. The small number of patients in both studies is a limiting factor to draw any conclusion. The PROSPECT trial (NCT01515787) is ongoing and should help further clarify the role of neoadjuvant systemic chemotherapy alone. If organ preservation is sought, radiotherapy should currently be included in the treatment strategy.

### High-Dose RT/Brachytherapy

We know that there is a strong correlation between dose of radiotherapy and tumour response. In rectal cancer, the work of Ann Appelt has helped to quantify it [53]. Her team analysed the data on tumour regression from 222 patients with rectal adenocarcinoma, treated with nCRT, and derived a dose-response relationship. Tumour regression was defined using the Mandard system. Total tumour dose was calculated by adding the brachytherapy component of the treatment to the external radiotherapy dose. ‘Complete response’ was defined as TRG1, and ‘major response’ was defined as TRG1-2. They found a strong correlation between total tumour dose and tumour response, with 92.0 Gy being the dose resulting in 50% of response for TRG1, and 72.1 Gy for TRG1-2.

One way of escalating the RT dose is with endorectal brachytherapy. One of its advantages over external beam RT is that it can deliver high dose of RT to a localised area. The exposure to surrounding healthy tissues is reduced, as the dose decreases with distance from the source. Buckley and colleagues did a systematic review on high-dose-rate brachytherapy (HDRB) in rectal cancer [54]. They investigated the role of HDRB alone, HDRB combined with CRT and HDRB in the context of W&W. The weighted mean rate of pCR with preoperative HDRB alone was 23.8% and with preoperative HDRB combined with CRT, 22.2%. The main limitations of this systematic review were the large variations in patient selection, the lack of long-term outcomes and the significant paucity of data on toxicities. Only one study used HDRB in a context of W&W and reported good outcomes [55•]. The treatment administered included CRT (60 Gy external beam RT with combined tegafur-uracil) followed by a 5-Gy HDRB boost. Seventy-eight per cent of patients achieved cCR, and 25.9% of patients experienced local relapse but all were successfully salvaged. Te Vuong and her team at McGill University have accumulated years of experience in HDRB and have reported good oncological outcomes in selected patients [56, 57]. The CORRECT trial (NCT02017704) is an ongoing randomised study evaluating the effectiveness of HDRB compared to standard nCRT in rectal cancer.

Understandably, it makes sense to aim for dose escalation, knowing that it will likely provide higher tumour regression, but in rectal cancer, caution is advised as the majority of patients will still go to surgery. It therefore might be inappropriate to push the dose above a certain level. Patients could face more complex surgeries, with a higher risk of complications and could potentially have to deal with poorer functional outcomes in the long term.

## Timing of Assessment

There is an ongoing debate on the best interval between treatment completion and response assessment. In the context of W&W, the objective is finding the perfect balance between greatest tumour regression, therefore increasing the patient’s chance of being eligible for a W&W approach, while assuring a safe and successful surgery, if surgery is inevitable. In the studies reporting on W&W, another serious limitation is the large variation on when these patients were identified and how. Some series described reassessment at a fixed time point, like 6, 8 or 10 weeks [55•, 58–62]. Others reported on response reassessment undertaken during a rather large period of time, for example 8 to 12 weeks, 4 to 10 weeks or simply more than 8 weeks without further details [63–66]. In order to clarify the optimal timing for restaging after neoadjuvant treatment, we can take a look at what we know so far on timing of surgery and its relation with the pCR rate.

Several retrospective studies have suggested a higher rate of pCR when delaying surgery after nCRT [67–69]. Since then, multiple prospective trials have been conducted to answer the question on timing, but report conflicting results.

Garcia-Aguilar and colleagues published in 2011 the initial results of a non-randomised phase II trial in which patients with stage II-III rectal cancer receiving nCRT pursued either TME at 6 weeks post treatment, or were given 2 cycles of consolidation mFOLFOX-6 followed by TME a further 3–5 weeks after completion of treatment [70]. The average time interval between the last fraction of radiotherapy and surgery was 6 weeks in the TME at 6 weeks group and 11 weeks in the delayed group. The surgeries were equivalent in both groups, without a significant increase rate of complications in either group. The pCR rate, on the other hand, was higher in the mFOLFOX-6 group compared to the standard group (25 vs 18%) although the difference was not statistically significant. The same group of investigators updated their result fairly recently, also adding a further 2 groups: one with 4 and one with 6 cycles of consolidation mFOLFOX-6 [71]. All patients underwent surgery at 3–5 weeks following the last cycle of chemotherapy. Automatically, this led to each group with a longer interval between nCRT and surgery: 8.5, 11.1, 15.4 and 19.3 weeks. The rate of pCR increased with the number of cycles of chemotherapy, from 18% in the standard nCRT group, to 25% in the 2 cycles of mFOLFOX-6 group, 30% in the 4 cycles group and finally 38% in the 6 cycles group. It is reasonable to believe that both the timing and the addition of consolidation chemotherapy had an impact on the pCR rate, but it is impossible to quantify the role of each.

The French Greccar-6 trial randomised 265 patients with cT3-4Nany or cTanyNpositive rectal adenocarcinoma, who completed nCRT, between surgery at 7 and 11 weeks post completion of treatment [72••]. In that study, increasing the interval between nCRT and surgery did not lead to a significant difference of pCR rate (15 vs 17.4%, *p* = 0.5983). Furthermore, increasing the interval to 11 weeks correlated with a higher risk of morbidity and a more complex surgical procedure. There were no significant statistical differences in the rate of anastomotic leak, perineal healing problems and mean hospital stay between the two groups. As opposed to the Greccar-6 trial, other studies did not show any negative impact of delaying surgery on the surgical outcomes and complications/morbidity rates [73, 74].

A similar trial on timing of surgery was conducted in the UK [75••]. In the 6 vs 12 study, 237 patients were randomised between surgery at 6 versus 12 weeks post nCRT. More patients in the 12-week interval group showed tumour downstaging (58 vs 43%, *p* = 0.019). The pCR rate was also significantly higher in the 12-week arm (9 vs 20%, *p* < 0.05). The results were presented in an oral abstract at the European Society of Medical Oncology (ESMO) annual meeting in Copenhagen in October 2016, and the final report is awaited.

Sun et al. interrogated the US National Cancer Database (NCDB) to answer the question on optimal timing [76]. The database was questioned on patients treated between 2006 and 2012, who received nCRT followed by surgery for a stage II–III rectal adenocarcinoma. The analysis included 11,760 patients. The investigators looked at the time between last fraction of radiotherapy and date of surgery, with endpoints including resection margin positivity, pathological downstaging, unpredicted postoperative readmission within 30 days, 30 day mortality rate and overall survival. They also aimed at finding an optimal time threshold for surgery (lowest R1 resection rate while highest proportion of tumour downstaging), which was found at 56 days (8 weeks) post nCRT. The authors found out that tumour downstaging increased during the waiting period, but passed 56 days, there was no added benefit of delaying surgery. Similarly, the risk of an R1 resection was stable until 56 days, but passed that time it increased significantly. Interestingly, the investigators did not find any threshold regarding readmission rate, 30-day mortality rate and overall survival. However, when looking at patients in the group who had surgery after the 56 days threshold, adjusted and compared to the group < 56 days, patients in the > 56 days group seemed to have a lower rate of readmissions, same mortality at 30 days, but worse overall survival (hazard ratio 1.2). Another group of investigators has also interrogated the NCDB, over the same period (2006–2011), and came with different results [77]. They divided patients between 3 groups depending of the time interval between nCRT and surgery: < 6 weeks, 6–8 weeks and > 8 weeks. 17,255 patients were included in the analysis. The > 8-week interval showed the best rate of pCR and downstaging, and patients had lower rates of 30-day readmission. Surgery in the > 8 weeks group was not associated with more complications or morbidity.

Petrelli and colleagues conducted a meta-analysis on timing between nCRT and surgery, with the pCR rate as primary endpoint [78•]. Thirteen studies, of overall medium quality, were included in the analysis. Investigators divided results into 2 groups: surgery at < 8 weeks and surgery at > 8 weeks. The longer interval led to a higher rate of pCR (RR 1.42, *p* < 0.0001). There were no significant difference in the overall survival, disease-free survival, rate of complete resection (R0), rate of sphincter preservation procedure and wound and anastomotic leak events.

Despite several good quality studies and best efforts, the optimal interval between nCRT and surgery, and similarly between nCRT and response assessment in a W&W context, has still not been established. In the absence of stronger evidence towards a precise time point, clinicians who are considering W&W for their patients should organise response assessment investigation between 6 to 12 weeks after completion of treatment and restrain from the impulse of proceeding to surgery on those who do not show cCR at this stage. Patients who have shown some degree of response might actually benefit from longer waiting and perhaps consolidation chemotherapy in some of them.

## Identification of Response

One of the major challenge in the NOM of rectal cancer is the careful selection of patients suitable for this approach. The objective is being able to identify patients who would have a pCR if operated on, out of all those who achieve a cCR. For the moment, the methods employed have several limitations, and cCR does not correlate perfectly with pCR. In the original publication from Habr-Gama and colleagues, 8.3% of the patients classified as incomplete responders had in fact a pCR on examination of the final TME specimen [10]. Other groups have reported even greater discordance. Creavin and colleagues showed that 15.9% of the patients in their cohort, after having nCRT and being considered partial responders, had a pCR [59]. In Maas and colleagues’ cohort of patients, out of 20 patients with a pCR, 15 of them had suspicion of residual disease on imaging exams post nCRT [79].

### Biopsy

At the time of response assessment, especially in the context of W&W, many clinicians are concerned about residual disease and routinely proceed with biopsy. Even in the absence of any residual mucosal abnormality, some people will proceed with random biopsies of the treated scar area, with the hope of being able to document a complete response. When a visible abnormality is suspicious for residual disease, some people would also proceed with biopsy, in that case to confirm the presence of tumour and support the indication for surgery. The São Paulo group did a retrospective comparative study to determine the value of biopsies post nCRT [80]. They reported a sensitivity of 50% and a poor negative predictive value of 11%, which might be explained by geographical miss. The specificity and the positive predictive value were both 100%, meaning that when cancer cells were found on the biopsy, it resulted in confirmed residual cancer in the resected specimen in all cases. These numbers are derived from only three biopsies truly negative and therefore should be taken cautiously. Nevertheless, some people will say that a positive biopsy is enough evidence on its own to proceed with surgery. We disagree, and question the justification of surgery on the basis of a few cells discovered in the sample, without any way of proving their viability. The aspect of timing is also crucial here. A biopsy taken at 6 weeks might be completely different than one taken at 12 weeks. We know that tumour regression continues beyond 8 weeks, but what if it is still the case after 12 weeks? That is the reason why we strongly favour the concept of regrowth. A biopsy reflects the situation at a precise time point. In our opinion, monitoring for regrowth, using imaging modalities and clinical examinations, for example, is a more reliable measure as it takes into account the notion of time. If there is evidence of regrowth, than the residual cells have proven their viability and their ability to proliferate. That is when an intervention, in most cases salvage surgery, should be considered, but a randomised trial is needed if such an approach is to be adopted to ensure that patients are not disadvantaged during this wait period through the development of metastatic disease.

### Transanal Endoscopic Microsurgery (TEM)

In order to maximise the chances of organ preservation, some investigators have described a new entity, the ‘near complete response (nearCR)’. It usually refers to tumours that show a very good response to treatment, but do not fulfil all criteria for a cCR. When a patient presents a nearCR, some people would favour TEM when technically feasible and have described good rates of organ preservation with this approach [59, 62]. Proceeding with TEM avoids the geographical miss that is possible with random biopsies, but timing is still an issue. In a study by Martens et al., patients who achieved a nearCR after nCRT were offered the choice between reassessment in a further 3 months, versus TEM [60]. Thirty-nine patients presented with a nearCR. Fifteen of them opted for TEM, amongst which 9 (60%) had confirmed pCR. All the remaining 24 patients who opted for further reassessment eventually achieved a cCR and entered the W&W program. The CARTS study was a prospective multicentre trial in which patients with early-stage cancer, eligible for TEM, underwent the procedure after nCRT [81]. The aim of the study was to accurately determine the number of patients with minimal residual disease (ypT0-1). Out of 51 patients who completed nCRT (50–50.4 Gy in 25–28 fractions with concomitant capecitabine), 47 experienced significant tumour regression (ycT0-2) and underwent TEM. There were 21 patients showing pCR (ypT0N0), 9 patients with ypT1N0 and 17 patients with ≥ypT2 and/or N+ disease. Twenty-eight per cent of patients who had TEM experienced postoperative complication. On top of the inevitable risk of overtreatment, TEM after CRT may result in poorer functional outcomes [14•, 24, 82•]. TEM should only be considered in a very selective group of patients following nCRT. The 21 patients with pCR in the CARTS study were obviously overtreated, and we believe that some of the patients with residual disease would probably have pCR as well, if allowed more time to achieve it. In the study, TEM was undertaken 8–10 weeks following the last fraction of RT.

### DRE/endoscopy

**Fig. 2:**
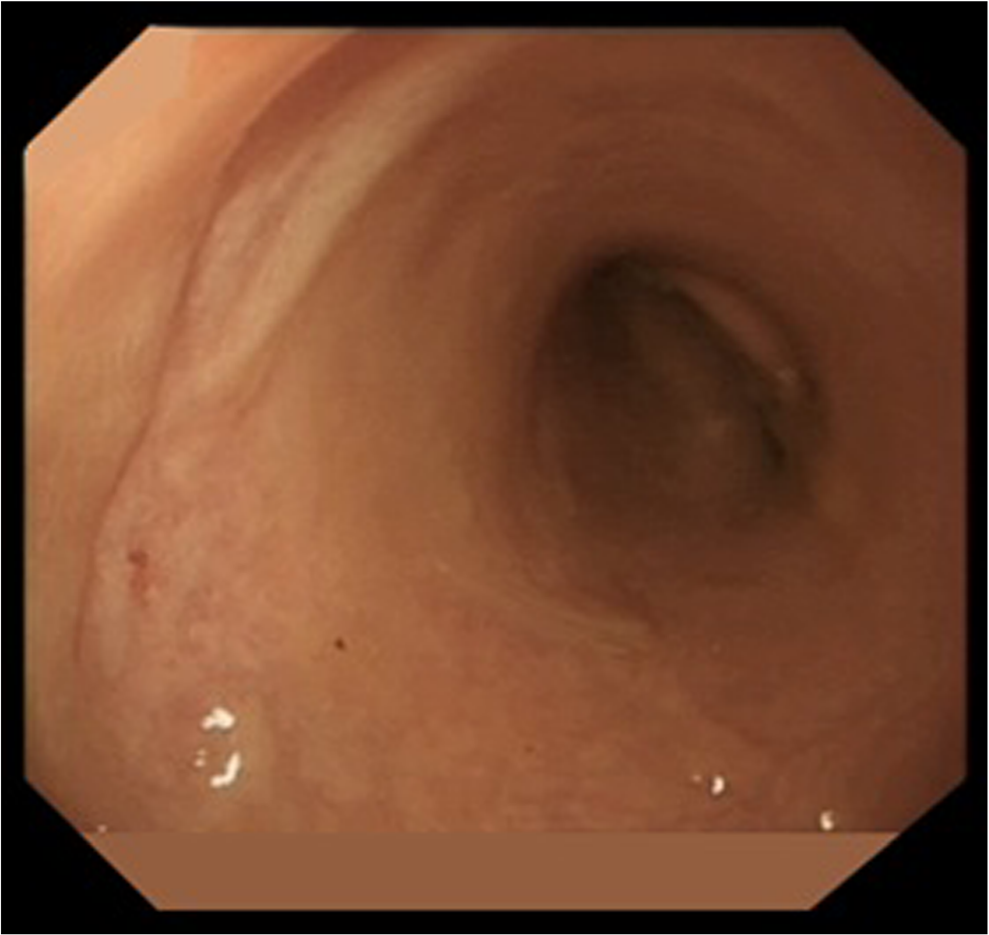
Maintained complete response of a rectal tumour, 4 years following chemoradiation. A typical appearance scar is visible, with no evidence of tumour recurrence

The definition of cCR varies between published series on W&W, but the most commonly used is the one proposed by Habr-Gama and colleagues [83]. On endoscopic and clinical evaluation, a whitening of the rectal mucosa can be observed, with or without associated telangiectasia (Fig. 2). The rectal wall can also show loss of pliability. Possible signs of incomplete response are, on endoscopy, the observation of any residual ulcer (deep or superficial, with or without a necrotic centre), significant stenosis and on digital examination the palpation of any residual nodule.

Most, if not all, series on W&W have included DRE and endoscopy as part of the reassessment protocol. We agree that those are helpful tools; however, they are not sufficient on their own. More prominent fibrotic changes could be easily interpreted as residual disease at the site of the primary tumour. Therefore, imaging might be a more robust and less subjective method of assessing response.

### PET-CT

Positron emission tomography and CT (PET-CT) is an imaging modality actively studied in the context of treatment response assessment. Given the fact that a metabolic complete response does not indicate pCR in all cases, a number of factors are being looked at to increase the value of PET-CT in a W&W context: a reduction in the maximal standardised uptake value (SUV), the total lesion glycolysis (TLG) and its percentage change and the metabolic tumour volume (MV), for example. In a series by Perez et al., the accuracy of PET-CT to predict cCR was 91% and increased to 96% when combined with clinical assessment [84].

### MRI

**Fig. 3:**
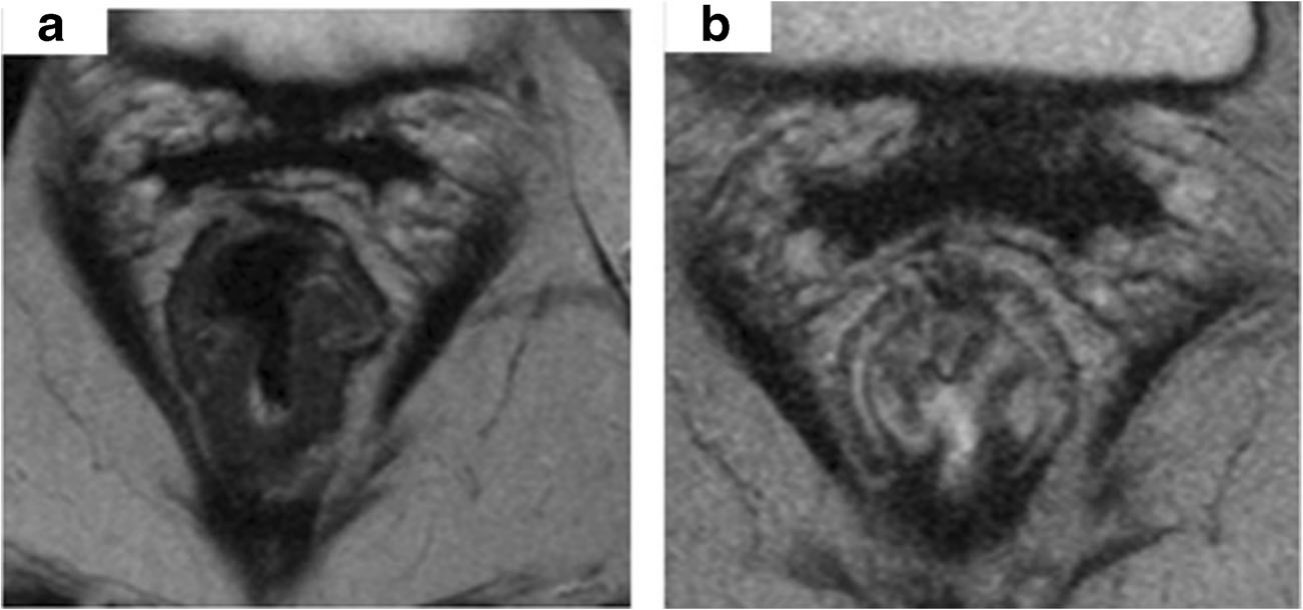
Low rectal tumour before treatment with full invasion of the muscularis at 4–8 o’clock position. The tumour borders the intersphincteric plane, therefore the CRM is involved at the level of the distal levators (**a**). Post CRT MRI demonstrates an area of low signal fibrosis at the site of the treated tumour, and no evidence of macroscopic residual intermediate signal suggestive of tumour—mrTRG2 (**b**)

**Fig. 4:**
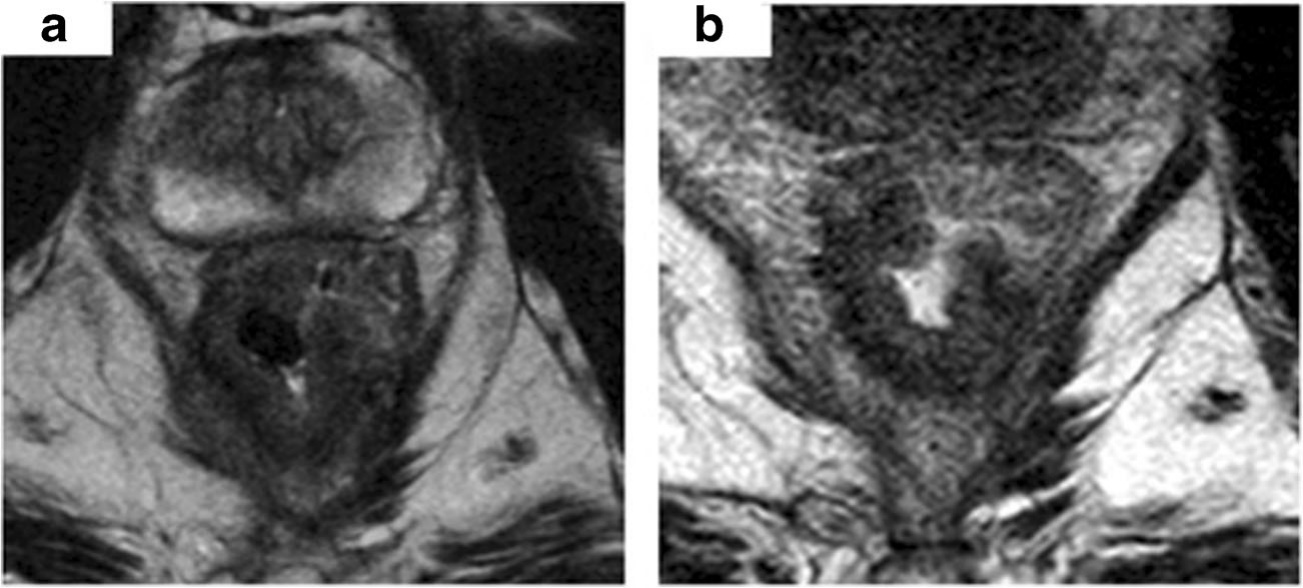
Low rectal tumour before treatment, infiltrating the rectal wall at 5–10 o’clock position with evidence of spread beyond the muscularis and spread into the intersphincteric plane, with invasion of the right levator (**a**). On post CRT MRI, the treated tumour demonstrates intermediate signal predominantly suggestive of macroscopic residual disease—mrTRG4 (**b**)

Magnetic resonance imaging (MRI) is a powerful tool to assess tumour response. There has been great interest in two different sequences of MRI: the standard MRI (mostly T2-weigthed sequence to assess changes within the treated tumour) and the diffusion-weighted MRI (DW-MRI). In any case, MRI should be of high quality to enable visualisation of fine details of the tumour spread as well as assessment of tumour response. For the last decade an MR-modified Mandard grading system (mrTRG) has been used to identify fibrosis/residual tumour signal and categorise patients in groups depending on the qualitative changes within the treated tumour [85]. mrTRG has also been proven to correlate with ypT and be a predictor of outcomes in rectal cancer patients [86]. According to mrTRG grading system patients are categorised in 5 groups:

- TRG1—thin fibrosis, low-density signal on T2-weighted images with no evidence of intermediate signal intensity at the site of the treated disease
- TRG2—dense fibrosis with no macroscopic evidence of intermediate T2 signal intensity (Fig. 3)
- TRG3—predominating low signal fibrosis with macroscopic scattered or local intermediate signal intensity
- TRG4 and TRG5—predominating intermediate T2-weighted signal with minimal or no fibrosis present (Fig. 4)

This imaging method of assessment response has been tested in a number of international prospective trials and confirmed to be an independent factor of prognosis and also shows to be a reproducible imaging tool [87]. Furthermore, a recent analysis showed that mrTRG is 10 times more likely to identify patients with complete pathological response when compared with clinical assessment, particularly residual mucosal abnormality [88]. A number of investigators have been particularly interested in the diffusion-weighted (DW) imaging proposing that this method could improve specificity of patients’ selection with complete response. Supporters of the DW imaging approach are suggesting that residual high signal intensity at the site of the treated tumour on high b-values images is indicative of areas of residual tumour and these patients should not be offered deferral of surgery approach. However, results of the Maastricht group work showed that clinical assessment with additional DWI missed 15% of patients with complete pathological response [89]. Currently, there is no evidence to indicate that apparent diffusion coefficient (ADC) measurements improve accuracy in detecting patients with a good response to treatment or that it provides any added value when mrTRG is utilised and studies have not yet been able to validate cut-off values or definitions for good versus poor response using DWI or ADC values that can be prospectively tested against survival outcomes [90–92].

Results of the Deferral of Surgery trial suggest that use of DWI protocols and PET/CT would have excluded over 30 and 60% of patients respectively with no regrowth for at least 1 year. Therefore, mrTRG appears to be the most sensitive method of finding patients with pCR not compromising the regrowth rates when compared with clinical assessment and DWI, PET/CT findings. This data was presented in an oral abstract at the European Society of Gastrointestinal and Abdominal Radiology (ESGAR) annual meeting in Prague in 2016, and the final report is awaited.

### Other Imaging Modalities

The modalities discussed above, namely clinical assessment, MRI and PET-CT, are the most frequent tools used in a W&W management. Endoscopic ultrasound, although proven valuable for the initial staging of rectal cancer, is unfortunately not the most helpful in tumour reassessment post treatment. The images are often distorted and even an experienced eye may struggle in differentiating residual tumour from chemoradiation change [93•]. Similarly, the standard CT scan, due to poor soft tissue contrast, has shown low accuracy of less than 50% to assess and predict pCR [93•].

**Fig. 5:**
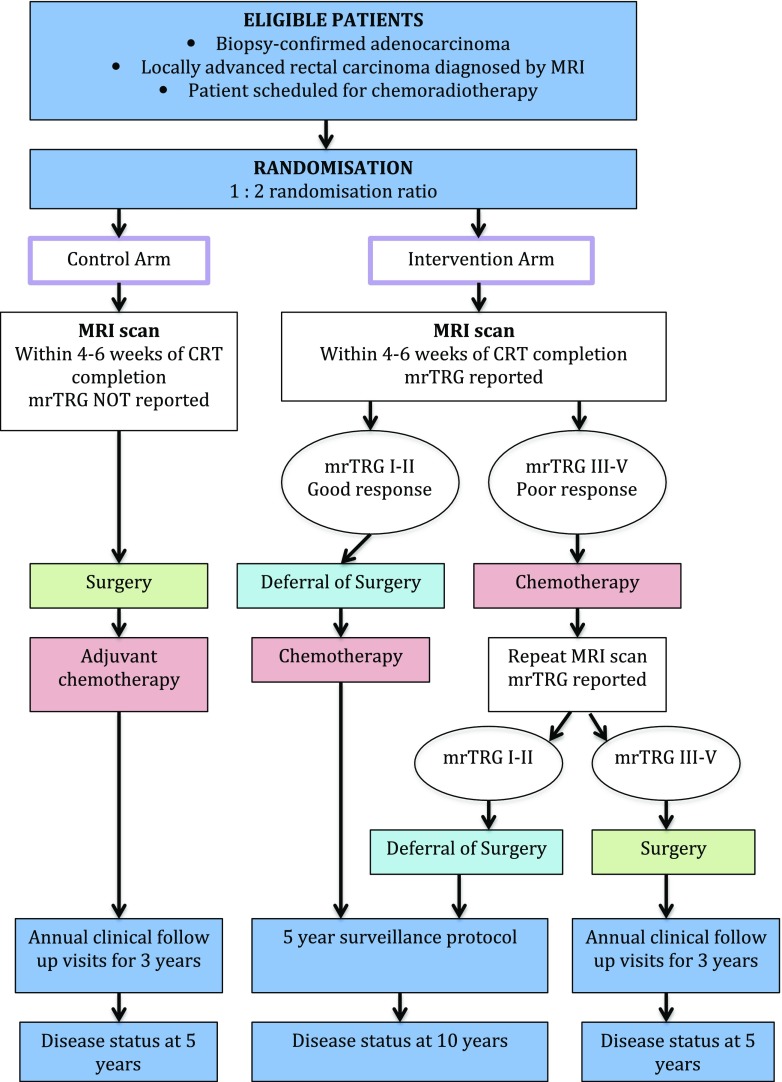
TRIGGER trial flowchart (summary). Further details are available in the protocol, including chemoradiotherapy regimen, chemotherapy agents and number of cycles

The published series on W&W have all used different modalities to assess response post nCRT. The MSKCC has created a three-tiered evaluation plan (The Regression Schema) that includes endoscopy, DRE, T2 weighted MRI and DW-MRI. The findings defined for each modality are used to divide patients between complete responders, near complete responders and incomplete responders. This assessment protocol is currently being tested and validated in the MSKCC phase II multi-institutional trial mentioned earlier [49]. At our institution, we favour mrTRG and are validating this biomarker in the TRIGGER feasibility trial [94••]. In the interventional arm of the trial, the patient is classified as either ‘good’ or ‘poor’ responder, based on mrTRG only. We do not take into account DRE and endoscopy findings at this point. Good responders undertake 12 weeks of consolidation chemotherapy and enter a surveillance program (deferral of surgery arm). Poor responders receive 12 weeks of consolidation chemotherapy before being reassessed with a further MRI. Patients who then fulfil the criteria for good responders enter the same surveillance program. Patients still deemed poor responders are referred for radical surgery. Endoscopy and DRE are used in the surveillance protocol, and their findings are taken into account when there is suspicion of regrowth. The trial flowchart is summarized in Fig. 5.

## What Is the Evidence that We Can Predict Response/Risk of Regrowth from Baseline Characteristics?

Several investigators have tried to determine and better understand factors that may predict response to CRT. This is an attractive field of research, as it has the potential to help clinicians select the appropriate candidates for organ preservation. A group of investigators has developed a nomogram to predict pCR post nCRT in locally advanced rectal cancer [95]. It includes as predicting factors pre- and post-treatment CEA level, distance from anal verge, circumferential extent of tumour and tumour size, and the authors report a 75–80% accuracy of the model to predict pCR. Other researchers have developed similar nomograms, using different factors [96, 97]. This is interesting and could indeed give us an idea of the likelihood of achieving pCR, but these nomograms will always remain imperfect. There are still so many confounders that have the potential to influence the tumour response and possibly regrowth for those patients managed with W&W.

### Tumour-Related Factors

Rectal cancers represent a group of diverse tumours, with inherent characteristics that can potentially influence the outcome of the treatment. Histology is one of them. The vast majority of tumours are adenocarcinoma, but subtypes include mucinous cancers which come with poorer outcomes and relative resistance to CRT [98]. Also, tumours that macroscopically look similar can express a wide range of different mutations. Furthermore, there is intratumoral heterogeneity, which illustrates the danger of supporting a management strategy and predict pCR on a biopsy sample only. The São Paulo group studied this concept, and by looking at somatic mutations in different fragments of a same tumour, found that the majority of the mutations (60%) were present in only one fragment, with only 27% of mutations being expressed in all fragments [99]. Similarly, two rectal cancer patients with the same overall burden of disease can express different carcinoembryonic antigen (CEA) levels. Several studies have found the CEA level, either prior to any treatment or after nCRT before radical surgery, to be a strong factor of tumour regression and pCR [100–102]. The location of the tumour within the rectum, its size, its circumferential extent, have all been recognised as possible predictive factors of response [103, 104].

### Patient-Related Factors

There are many characteristics of the host that could potentially influence the outcome of the treatment. Examples include the immune system, the presence of chronic disorders such as inflammatory bowel disease or diabetes and active cigarette smoking. Medication might be an important confounder, for example immunosuppressor agents like cyclosporin, tacrolimus or even simple corticosteroids. There is not much data on medication and response to treatment, but researchers have found that the use of statins increases tumour regression with CRT [105–107]. There are at least two phase II trials currently running that investigate the value of adding a statin to nCRT in rectal cancer (NCT02161822 and NCT02569645). Similar to the CEA level, the link between the neutrophil-lymphocyte ratio (NLR) or other inflammatory markers and tumour regression is being studied and is promising [108–110].

### External Factors

The treatment administered, including the modality, the dose, the delays or interruptions, obviously has an impact on the outcomes. Timing, discussed in details earlier, has also a major influence on the response to treatment. Other possible confounders, on which there is paucity of data, include other interventions, like trauma from multiple biopsies, for example. Could it have an impact on the risk of regrowth?

## Monitoring of Patients

There are differences between monitoring residual disease and monitoring a cCR. As discussed above, at our institution, we favour the surveillance for absence of regrowth, irrespective of residual tumour. We have said earlier that, from our point of view, biopsies are unnecessary and we also believe they might actually be harmful. Unless we find a way of proving viability of tumour cells, waiting and monitoring for regrowth is preferable. The TRIGGER trial, discussed earlier, allows patients who have shown a good response, but in whom the possibility of residual tumour is not excluded, to be further reassessed and monitored for regrowth [94••]. Some people are sceptical of this approach and argue that there is a risk of distant metastasis with residual tumour. However, the data on W&W has shown good outcomes, including rate of distant metastasis (oncological outcomes are discussed further below). We agree that there is a lack of long-term data on W&W, including especially the risk of late distant recurrences, and that is why a randomised controlled trial such as TRIGGER provides a safer environment to investigate this treatment strategy.

The surveillance protocol in a NOM approach differs in the studies published so far, but like the identification of response process, it usually includes a combination of clinical examination, monitoring of CEA level, flexi-sigmoidoscopy and/or complete colonoscopy and imaging exams. No clear recommendation as to the best surveillance program has been defined, but several series have reported that the vast majority of regrowths occur in the first 2 years after completion of treatment. Therefore, most clinicians agree that until better understanding of the disease and more accurate predictors of relapse are known, the monitoring protocol should be intensive, at least for the first 2 years. Most groups of investigators have opted for clinical visits and exams every 1–3 months for the first 2 years. Consequently, patients who are being offered W&W should be fully aware of the implications of such an intensive program and should be given a summary of the surveillance schedule. Patients on whom the clinician has a doubt regarding compliance might not be good candidates for a W&W strategy, and clinicians should raise their concerns with their patients when that is the case.

The Deferral of Surgery trial (NCT01047969), a prospective study of patients managed with W&W in a controlled surveillance program, has been running at the Royal Marsden Hospital, in the UK, and recently completed recruitment. Its follow-up schedule is summarised in Table 1. Of note, biopsies should not be done routinely, but only if regrowth is suspected and should be taken only after MRI and PET-CT are performed to reduce the rate of false-positive finding on imaging. These proposed guidelines could be useful for clinicians who wish to put in place a W&W program at their own centre.

**Table 1:** Follow-up schedule in the Deferral of Surgery trial

Timeline from end of CRT	DRE	CEA	Scans	Endoscopy
4–8 weeks	✓	✓	MRI	
8–12 weeks	✓	✓	MRI, FDG-PET	
16 weeks	✓	✓	MRI, FDG-PET	
6 months	✓	✓	MRI	Flexi-sigmoidoscopy
9 months	✓	✓	MRI	Flexi-sigmoidoscopy
12 months (1 year)	✓	✓	CT, MRI, FDG-PET	Colonoscopy
15 months	✓	✓		
18 months	✓	✓	MRI	Flexi-sigmoidoscopy
21 months	✓	✓		
24 months (2 years)	✓	✓	CT, MRI	Flexi-sigmoidoscopy
30 months	✓	✓		
36 months (3 years)	✓	✓	CT, MRI	Flexi-sigmoidoscopy
42 months	✓	✓		
48 months (4 years)	✓	✓	MRI	Flexi-sigmoidoscopy
54 months	✓	✓		
60 months (5 years)	✓	✓	MRI	Colonoscopy
72 months (6 years)	✓	✓	MRI	Flexi-sigmoidoscopy
84 months (7 years)	✓	✓	MRI	Flexi-sigmoidoscopy
96 months (8 years)	✓	✓		
108 months (9 years)	✓	✓		
120 months (10 years)	✓	✓		Colonoscopy

*CRT* chemoradiation treatment, *DRE* digital rectal examination, *CEA* carcinoembryonic antigen

## Quality of Life

Since the NOM approach in rectal cancer is fairly recent, little is known regarding the functional outcomes and quality of life of patients being managed with this strategy. Some authors have attempted comparing it with anal cancer, in which the first-line treatment is definitive chemoradiation, but such a comparison is inadequate. This is mostly due to the different chemotherapy regimen, radiation fields, doses and dose distribution.

Only few publications on W&W have reported quality of life (QoL) outcomes. Appelt and colleagues did a prospective observational study on patients with cT2-3N0-1 resectable rectal adenocarcinoma between 0 and 6 cm from the anal verge, treated with nCRT [55•]. The radiation dose was higher than standard, with the gross tumour receiving up to 60 Gy by external beam radiotherapy, followed by a 5-Gy boost by endorectal brachytherapy (equivalent total dose on tumour 66 Gy). The concomitant chemotherapy was oral tegafur-uracil. Patients with evidence of cCR following nCRT entered an active surveillance protocol. Investigators assessed toxicities, function and QoL using The European Organisation for Research and Treatment of Cancer (EORTC) colorectal cancer-specific QoL module (QLQ-CR29) and the Jorge-Wexner scale for faecal incontinence. The EORTC questionnaire was completed prior to start nCRT, at the end of treatment, at 6 months post treatment, 12 months post treatment and on a yearly basis thereafter. Faecal incontinence was recorded at every visit. A total of 40 patients formed the observation group (W&W). Eighteen of 25 patients (72%) reported no faecal incontinence at 1 year and 11 of 16 (69%) at 2 years. The EORTC scores remained quite similar during the study period. The median score at baseline (38 patients) was 9.7, at 12 months 10.1 and at 24 months 13.8. The most frequent late toxicity reported was rectal bleeding (78% of patients), of mild severity in the majority of patients.

As mentioned in a previous section, Martens and colleagues prospectively studied a cohort of patients who received standard nCRT, achieved a cCR and were consequently offered an organ preservation strategy [60]. Patients with a cCR followed an active surveillance schedule, whereas patients who had a nearCR had two choices: to proceed with transanal endoscopic microsurgery (TEM) or to continue with surveillance in a further 3 months, at which point decision would be made between W&W and TME. Faecal incontinence was assessed using the Vaizey score. Patients with a minimum of 3 years of follow-up were offered to complete the questionnaire. A total of 100 patients entered the program. There were 61 patients with an initial cCR who followed the W&W surveillance protocol, 24 patients with a nearCR who ended in the W&W program after a second assessment confirmed cCR and finally 15 patients with a nearCR who underwent TEM. Forty-five patients with no evidence of regrowth and at least 3 years of follow-up were approached to complete the Vaizey questionnaire, which measures faecal incontinence. Twenty-nine patients agreed to complete it (22 W&W and 7 TEM). Patients in the W&W group reported a better continence, with a mean score of 3.4, compared to the TEM group with a 9.7 mean score (*p* = 0.003). One patient in the W&W group, and 3 in the TEM group, reported major incontinence, defined as a score above or equal to 12. The same group of investigators published earlier in 2011 the initial results of their pilot study, in which they measured the functional outcome of 21 patients in a W&W program [79]. They used the MSKCC bowel function questionnaire and assessed faecal incontinence with the Wexner incontinence score. Results were compared with those of a cohort of patients having pCR following nCRT and TME. Patients in the W&W group showed better bowel function score and lower incontinence score compared to the pCR group: they seemed to be less affected by food intake, were less likely to use pads, had better control over flatus and reported less changes in their bowel habits.

Habr-Gama and colleagues also evaluated the anorectal function of their cohort of patients managed with W&W (cCR patients) or TEM (nearCR patients) [82•]. The functional outcome was assessed at least 8 weeks after treatment in the W&W group and at least 6 months after the procedure in the TEM group. They used the Cleveland Clinic Incontinence Index (CCII), performed anorectal manometry with the ALACER manometer (Multiplex II) and finally evaluated quality of life with the Fecal Incontinence QoL Index Scale (FIQL). The results of all assessments were better in the W&W group compared to the TEM group. For the W&W group, results in each category of the FIQL were (4 being an optimal score): lifestyle 3.5, coping/behaviour 3.4, depression/self-perception 3.4 and embarrassment 3.5, whereas in the TEM group results were, respectively, 3.0, 2.7, 3.1 and 3.1. The CCII score was 2.3 in the W&W group, compared to 6.5 in the TEM group.

In the Deferral of Surgery trial, bowel function and QoL data have been prospectively collected, using the LENT/SOMA system for acute and late toxicities, the EORTC QLQ-C30 Questionnaire, a Modified Inflammatory Bowel Disease Questionnaire and the Vaizey Incontinence Questionnaire. It will provide precious information on functional outcomes and QoL.

## Outcomes and Management of Regrowth/Relapse

Dossa and colleagues did a systematic review and meta-analysis on the safety and outcomes of a W&W approach in patients achieving cCR [111••]. They analysed the combined evidence on rate of local regrowth and salvage therapy and compared the rates of relapse and survival outcomes between patients managed with NOM and patients managed with surgery. They defined local regrowth as evidence of intraluminal tumour detected clinically, endoscopically or radiologically. Nodal disease was considered as non-regrowth recurrence, which also included any non-luminal intrapelvic disease or distant metastatic disease. The final analysis included 23 studies, with a total of 867 patients. The pooled rate of local regrowth was 15.7% (95% CI 11.8–20.1), and following a regrowth, the pooled rate of salvage therapy was 95.4% (95% CI 89.6–99.3). For those patients undergoing salvage surgery, the rate of sphincter preservation was 49.8% (95% CI 33.0–66.6). Compared to patients managed with surgery, patients being followed in a W&W protocol did not have any significant difference in non-regrowth recurrence, cancer-specific mortality and overall survival. Based on their results, the authors were able to estimate that out of 1000 patients managed by W&W, only 2 patients would not be candidates for salvage therapy given the extent of their locoregional disease, and one patient would not be a candidate because of the presence of metastatic disease.

Kong et al. also undertook a systematic review on the outcomes of the W&W approach [112••]. They measured the rate of local regrowth, salvage surgery, disease-free and overall survival. For their analysis, they considered tumour regrowth and local (rectal) recurrence as the same event for patients managed with W&W and named it tumour regrowth. They defined local recurrence as any evidence of relapse after surgery, either immediate surgery or salvage surgery. Nine studies met the inclusion criteria and were included in the final review, with a total of 370 patients in the W&W group. The rate of tumour regrowth was 28.4%, with a rate of salvage surgery of 83.8%. The rate of distant recurrence without tumour regrowth was 1.9%. The reasons for not undertaking salvage surgery was equal (about a third each) between: presence of distant metastases, patients unfit for surgery and patients declining surgery. The rate of distant recurrence was similar between patients in the W&W group and patients having immediate surgery. Disease-free survival and overall survival differed between studies, but it ranged from 97% at 2 years to 91% at 5 years for OS and 88% at 2 years to 68% at 5 years for DFS, without any significant difference when compared to patients having a pCR after immediate surgery.

In 2014, a group of expert within the Champalimaud Foundation and EURECCA (European Registration of Cancer Care) established the International Watch and Wait Database (http://www.iwwd.org). The objective of this project is to gather the evidence on W&W strategies, by combining the existing retrospective data and the data prospectively collected. In August 2016, the database included 775 patients, and the first report was presented by Dr van der Valk at the annual ASCO GI meeting in January 2017 [113•]. With a median follow-up time of 2.6 years, the rate of local regrowth was 25%, and 84% of these regrowths occurred in the first 2 years after treatment. Ninety-six per cent (96%) of the regrowths were intraluminal, and 4% were locoregional nodal recurrence. Seven per cent (7%) of patients developed distant metastatic disease. The OS at 3 years was 91%.

## Conclusion

The future is promising with regard to organ preservation in rectal cancer, and interest is growing fast. Despite some major advances in the last few years, there are still many challenges and aspects of the strategy that need refinement. The selection of patients, the identification of accurate predictors of response and the increase in response rates by intensifying treatment yet offering an acceptable toxicity profile are all examples of exciting areas of development. With the evidence we have so far, we could reasonably predict that the treatment of rectal cancer is likely to be tailored and risk-adapted to each specific patient. For example, patients who show some response to nCRT could be monitored and be allowed longer delays in order to let them achieve the best possible response. Some tumours might take more than 12 weeks to achieve cCR, and with a careful surveillance program, this approach should not compromise in any way the patient’s ultimate outcome. On the other hand, patients who do not respond to treatment are unlikely to benefit from longer waiting period and should proceed with surgery at a time point that allows a safe and successful procedure. Alternatively, they might be the ideal candidates for further chemotherapy.

Currently, there is no level I evidence to support a W&W approach in patients achieving cCR after nCRT for rectal adenocarcinoma. The aim of the International Watch and Wait Database is to provide the strongest available evidence on the W&W management strategy. However, this database seems to combine a heterogeneous group of patients, stages, assessments and monitoring protocols that may lead to inaccuracy of results. Moreover, one of the main concerns with W&W is the lack of long-term data, especially on patients who experience regrowth, to confirm the safety of this approach. A randomised controlled study like the TRIGGER trial will provide more reliable answers.
